# How does Conditional Regard Impact Well-being and Eagerness to Learn? An Experimental Study

**DOI:** 10.5334/pb.401

**Published:** 2018-04-27

**Authors:** Sofie Wouters, Sander Thomaes, Hilde Colpin, Koen Luyckx, Karine Verschueren

**Affiliations:** 1 KU Leuven, School Psychology and Development in Context, Faculty of Psychology and Educational Sciences, BE; 2 Department of Psychology, Utrecht University, NL

**Keywords:** conditional regard, self-esteem, affect, eagerness to learn, experiment

## Abstract

Conditional regard refers to regard dependent upon the receiver’s fulfillment of certain expectations. Using an experimental design, we examined the effect of conditional negative and positive regard on well-being and eagerness to learn in university freshmen (*N* = 131). Participants experienced either failure or success followed by conditional vs. unconditional regard. As expected, success and failure had opposite effects on well-being and eagerness to learn. More importantly, there was an increase in positive affect following success in the context of conditional regard, but not in the context of unconditional regard. Additionally, the decrease in positive affect following failure was more pronounced when accompanied by conditional as compared to unconditional regard. Conditional regard thus magnified the impact of success versus failure on students’ emotional experiences.

In the present study, we examined how students’ self-esteem, affect, and eagerness to learn may be impacted by others’ feedback. We focused on these outcomes as research has shown that higher self-esteem, more positive affect, and less negative affect are important indicators of student well-being (Kuppens, Realo, & Diener, 2008; Zeigler-Hill, 2013). Additionally, higher levels of well-being and an increased focus on learning have been associated with many beneficial educational outcomes such as higher achievement and more engagement (e.g., Cury, Elliot, Da Fonseca, & Moller, 2006; Pekrun, Elliot, & Maier, 2009). Specifically, we concentrated on a particular type of feedback, that is conditional regard. Conditional regard (CR) refers to regard that is dependent (i.e., conditional) upon the receiver’s compliance with another person’s expectations or demands. Previous research on CR, which has mainly focused on parental CR, has broadly demonstrated that CR may harm students’ well-being (Kanat-Maymon, Roth, Assor, & Raizer, 2015). Yet, extant research has primarily been correlational, and so the causal role of CR in well-being and motivation remains unclear. Therefore, we tested the effect of experimentally induced CR following success or failure on university freshmen’s self-esteem, affect, and eagerness to learn.

Scholars have distinguished between conditional *positive* and *negative* regard (i.e., CPR and CNR, respectively; Assor & Tal, 2012; Roth et al., 2009). In CPR, regard is provided or increased when the receiver meets one’s demands (e.g., providing (more) appreciation when a student is successful); in CNR, regard is withdrawn or decreased when the receiver fails to meet one’s demands (e.g., withdrawing or providing less appreciation when a student fails). How may CPR and CNR impact well-being and motivation? If students perceive that others’ valuation is contingent upon them meeting certain criteria (e.g., being successful academically or socially), they may internalize this and also come to value themselves based on these criteria. This may result in more contingent self-esteem, and hence, more fluctuations in self-esteem following success or failure (e.g., Lakey, Hirsch, Nelson, & Nsamenang, 2014; van der Kaap-Deeder et al., 2016). In other words, it may be expected that CR enhances the impact of success or failure on self-esteem.

With regard to CNR in particular, we expect it to negatively affect self-esteem, affect and motivation. Indeed, CNR may be considered a subcomponent of the broader construct of psychological control, also comprising guilt induction and shaming (Assor, Kanat-Maymon, & Roth, 2014). According to Self-Determination Theory (SDT; Deci & Ryan, 2000) psychological control is assumed to thwart adolescents’ needs for autonomy and relatedness and to result in negative affect towards its provider. CNR may be expected to thwart adolescents’ needs in similar ways and, thus, result in enhanced negative effects on self-esteem, affect and motivation following failure (Cordeiro, Paixao, Lens, Lacante, & Luyckx, 2016; Kanat-Maymon et al., 2015; Roth et al., 2009; Wouters et al., 2014). With regard to the effects of CPR, studies have shown retrospective reports of CPR in close relationships to have psychological costs (Assor et al., 2014; Kanat-Maymon et al., 2015). However, in the short term showing more personal appreciation after success may be expected to have (temporary) positive effects on the recipients’ self-esteem, affect, and motivation (Assor, Roth, & Deci, 2004).

Limited empirical evidence supports these hypotheses, showing that parental CNR is clearly detrimental with regard to students’ adjustment and motivation and that parental CPR is related to more shame or devaluation after failure, but also to more self-aggrandizement after success (Assor & Tal, 2012; Roth et al., 2009). Yet, these studies were correlational and relied on youth’s self-report of parental CR. To our knowledge, no previous study examined whether CNR actually enhances the effect of failure and CPR actually enhances the effect of success on students’ well-being and motivation, using an experimental design.

Nevertheless, there is some related evidence. For instance, one experiment showed that undergraduate students, who were primed to think about someone only accepting them on certain conditions (vs. those primed to think about an unconditionally accepting other), specifically associated failure on a lexical decision task with rejection, and success with acceptance (Baldwin & Sinclair, 1996). One other field experimental study asked secondary school students to imagine a situation in which they experienced unconditional regard (i.e., regard provided regardless of students’ behavior or performance), conditional regard, or another social event. This study found that lower grades (received three weeks after the manipulation) only resulted in more negative feelings for students who had *not* imagined unconditional regard (Brummelman et al., 2014).

Building on extant research (e.g., Assor et al., 2004; Assor & Tal, 2012; Baldwin & Sinclair, 1996; Brummelman et al., 2014), we designed an experiment to investigate the moderating role of CNR and CPR in the effect of performance on students’ self-esteem, affect and eagerness to learn. In the experiment, psychology freshmen were led to believe that they succeeded or failed on a digit span task (using manipulated false performance scores). Following success or failure, they received feedback implying conditional regard (i.e., regard that is conditional on performance) vs. unconditional regard. We measured state self-esteem and state affect at pre- and posttest to examine fluctuations in these outcomes. We measured students’ eagerness to learn as an indicator of their motivation at posttest.

We expected an increase in self-esteem and positive affect and a decrease in negative effect in the context of success and the reverse in the context of failure. Thus, we expected a significant two-way interaction between time and performance. In addition, we assumed these effects to be stronger in the context of conditional regard as opposed to in the context of unconditional regard. Thus, our main prediction was a significant three-way interaction between time, performance (success vs. failure) and regard (conditional vs. unconditional) for state self-esteem and affect. Specifically, in the context of failure, we expected that the decrease in self-esteem and positive affect and the increase in negative affect would be enhanced when the experimenter’s appreciation was lost based on this performance (CNR) (as compared to when unconditional regard was provided). Additionally, in the context of success, we assumed the increase in self-esteem and positive affect and the decrease in negative affect to be enhanced when the experimenter’s appreciation was clearly won based on this performance (CPR) (as compared to when unconditional regard was provided).

Similar hypotheses were formulated for differences in posttest eagerness to learn between success and failure conditions. Successful students are expected to be more eager to learn than failing students and this difference should be larger in the context of conditional vs. unconditional regard.

## Method

### Participants and Procedure

Data were collected in 2015 at a large European university. Participants were freshman university psychology students (*N* = 131; 82% female; *M* age = 19.27, *SD* = 1.69 years, range 17 to 29) who participated in exchange for course credit. Data from one student for whom the standard procedure was not strictly followed and one student older than 30 years were deleted. They were recruited via an online research enrollment interface. The experiment was approved by the institute’s ethical committee and informed consent was obtained from each student.

Participants were tested individually by a research assistant (i.e., one of two advanced female psychology students). At the start of the experiment, the experimenter told participants that the study would involve a training of short-term memory. She also stressed the importance of short-term memory abilities by describing these as predictors of future academic and career success. During the experiment, participants performed a digit span task and completed pencil-and-paper questionnaires (at pre- and posttest) in the presence of the experimenter. Students were thoroughly debriefed.

### Design

The experiment used a 2 (performance: success vs. failure) × 2 (regard: conditional vs. unconditional) between-subjects design. Participants were randomly assigned to conditions. Expecting medium-sized interactions (based on previous related research) and considering the current design and sample size, we expected to have adequate power (i.e., power ≥ .80). Participants completed a digit-span task created for the purpose of this experiment. The task required students to listen and immediately repeat each of the 20 digit-spans mentioned out loud by the experimenter. In the success conditions, students completed a relatively easy version of the task and received a score of 15 out of 20 (i.e., *success*). In the failure conditions, they completed a relatively difficult version of the task and received a score of 5 out of 20 (i.e., *failure*). These manipulated false scores were credible because the digit spans in the success vs. failure conditions were adjusted (i.e., they were made easier or more difficult) based on a pilot study.

Next, in the *unconditional regard conditions* (*n_success_* = 33; *n_failure_* = 33), the experimenter told participants, regardless of their (alleged) performance, that it was very important that they had participated in the training and also thanked them for participating. In the *conditional regard conditions*, feedback differed according to participants’ performance. In the conditional success condition (*n* = 32), the experimenter told the participant that he/she had done very well and that she liked working with him/her, especially because he/she had performed so well, thus showing personal appreciation based on the student’s performance. In the conditional failure condition (*n* = 33), the experimenter told the participant that he/she had not done well and that she had honestly expected a lot more, clearly showing the student had lost their personal appreciation based on the student’s performance. Thus, in the conditional regard conditions, a context was created in which relational valuation was coupled to performance, which is the core of conditionality. Both experimenters were trained to provide credible feedback during 10 try-outs in a pilot study.

### Questionnaires

To assess state self-esteem, we used four items from the Dutch version of the Rosenberg Self-Esteem Scale (RSES; Rosenberg, 1965; Van der Linden, Dijkman, en Roeders, 1983) adjusted to measure state self-esteem (e.g., ‘At this moment, I take a positive attitude toward myself’), using a 7-point scale ranging from 1 (*completely untrue*) to 7 (*completely true*).

State positive and negative affect were assed using the 20-item Dutch version of the Positive and Negative Affect Schedule (PANAS) state-version (Watson, Clark, & Tellegen, 1988; Engelen, De Peuter, Victoir, Van Diest, & Van den Bergh, 2006). Students indicated the extent to which they felt each of the 20 emotions at that given moment using a 5-point Likert scale ranging from 1 (*very little*) to 5 (*very much*) (e.g., ‘excited’ or ‘proud’ (positive affect) and ‘scared’ or ‘irritable’ (negative affect).

To assess eagerness to learn, students were asked if they wanted to take part in short-term memory training in the future, using a 3-point scale (0 = *no*, 1 = *maybe*, 2 = *yes*) at posttest. After completing the posttest questionnaire, participants were also asked to do a “surprise” extra training of 10 digit spans, for which they could choose their preferred difficulty level (ranging from 1 (*very easy*) to 7 (*very difficult*)), which served as our second measure of eagerness to learn.

### Manipulation checks

In the pretest questionnaire, students rated the importance of short-term memory abilities (3 items, sample item: ‘I think a good short-term memory increases your chances on academic success’, α = .68) on a 7-point scale ranging from 1 (*completely untrue*) to 7 (*completely true*). The three items measuring the perceived importance of short-term memory were averaged into one index.

After receiving feedback, students were also asked to evaluate their performance. The first item ‘I think I have performed … on the memory task’ was scored on a 6-point rating scale ranging from 1 (*very badly*) to 6 (*very well*); the second item ‘It feels like I failed on the memory task’ was scored on a 7-point scale ranging from 1 (*completely untrue*) to 7 (*completely true*) (reverse scored). These items were standardized and then averaged into a single index of performance self-evaluation (*r_Item1–Item2_* = .84).

Finally, to get an idea of whether the students had felt the personal appreciation or rejection of the experimenter in the conditional vs. unconditional regard conditions, we also asked about how kindly they felt treated by the experimenter (one item; ‘I feel that I am treated kindly by the student providing the training’) on a 7-point scale ranging from 1 (*completely untrue*) to 7 (*completely true*).

## Results

### Preliminary Analyses

Table 1 shows descriptive statistics for the study. Results indicate that self-esteem and positive affect were positively related to each other and negatively related to negative affect. At posttest, positive affect related positively to students’ intention to participate in future short-term memory trainings and both self-esteem and affect were also significantly related to the chosen difficulty level for the extra training. Finally, at posttest, students’ actual score on the memory task was significantly positively correlated with self-esteem, positive affect and chosen difficulty level and negatively with negative affect.

**Table 1:** Intercorrelations and Descriptive Statistics for all Study Variables.

	1		2		3		4		5		6		7	8		9

*Pretest*																
1. State self-esteem	—															
2. State positive affect	.32	***	—													
3. State negative affect	–.58	***	–.13		—											
*Posttest*																
4. State self-esteem	.69	***	.21	*	–.47	***	—									
5. State positive affect	.26	**	.59	***	–.07		.59	***	—							
6. State negative affect	–.50	***	–.10		.72	***	–.75	***	–.37	***	—					
7. Future participation	.08		.08		–.05		.15		.23	**	–.11		—			
8. Difficulty level	.04		.14		–.01		.26	**	.36	***	–.24	**	.07	—		
9. Actual score	–.11		–.01		.10		.35	***	.47	***	–.32	***	.16	.60	***	—
*M*	5.20		3.06		1.66		5.04		2.88		1.68		1.61	3.60		9.89
*SD*	0.99		0.54		0.57		1.18		0.77		0.72		0.52	1.07		4.61
*Cronbach α*	.85		.82		.86		.88		.90		.90		—	—		—


*Note*. * *p* < .05. ** *p* < .01. *** *p* < .001.

Furthermore, the mean of participants’ perceived importance of short-term memory (*M* = 5.35, *SD* = 0.84), was well above the midpoint of the scale being used (i.e., 4.00). A Performance × Regard ANOVA demonstrated that students in the success conditions rated their performance as significantly more positive (*M* = 0.84) than students in the failure conditions (*M* = –0.83), *F*(1,127) = 427.09, *p* < .001, partial *η^2^* = .77. A Performance × Regard ANOVA further showed a significant interaction between Performance and Regard on how students felt toward the experimenter, *F*(1,127) = 7.36, *p* < .01, partial *η^2^* = .06. The simple effects indicated that students in the conditional failure condition felt that they were treated significantly less friendly by the experimenter (*M* = 6.15) than those in the unconditional failure condition (*M* = 6.58), *F*(1,127) = 7.55, *p* < .01, partial *η^2^* = .06. Additionally, students in the conditional success condition felt that they were treated more friendly by the experimenter (*M* = 6.63) than those in the unconditional success condition (*M* = 6.46), but this difference was not significant, *F*(1,127) = 1.20, *p* = .28, partial *η^2^* = .01. We found no significant differences between conditions in state self-esteem, state positive and negative affect at pretest, indicating that random assignment to conditions had been successful.

### Main Analyses

**Figure 1:**
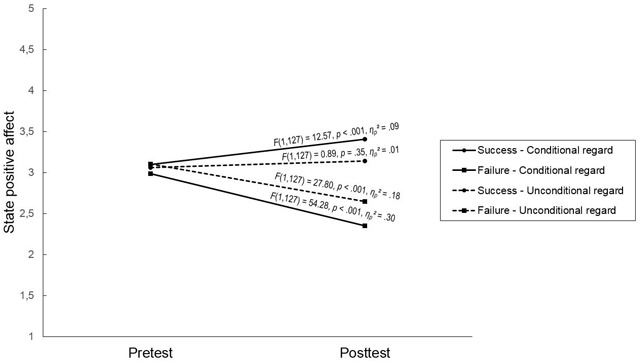
Three-way interaction between Time, Performance, and Regard on state positive affect.

Next, we tested our main hypotheses. All main findings were based on analyses without controlling for sex or age as covariates, as results were similar when controlling for these variables. First, we performed three separate Performance × Regard repeated measures ANOVAs on self-esteem and (positive and negative) affect. Students’ self-esteem and positive affect changed significantly over time, but these main effects were qualified by a significant Time × Performance interaction for all three outcomes, *F*_self-esteem_(1,127) = 77.04, *p* < .001, *η_p_^2^* = .38, *F*_positive affect_(1,127) = 73.25, *p* < .001, *η_p_^2^* = .37, *F*_negative affect_(1,127) = 67.03, *p* < .001, *η_p_^2^* = .35. Specifically, failing students experienced decreases in self-esteem and positive affect and an increase in negative affect, whereas the reverse was true for successful students. For positive affect, we also found a significant three-way interaction between Time, Performance, and Regard, *F*(1,127) = 5.61, *p* < .05, *η_p_^2^* = .04. As shown in Figure 1, failing students experienced more pronounced decreases in state positive affect when receiving conditional vs. unconditional regard (*η_p_^2^* =.30 vs. *η_p_^2^* = .18). Additionally, successful students experienced a significant increase in positive affect only when experiencing conditional regard, but not when receiving unconditional regard (*η_p_^2^* = .09 vs. *η_p_^2^* = .01). None of the other effects were significant.

In addition, two separate Performance × Regard ANOVAs on each of the posttest eagerness to learn indicators yielded a significant main effect for Performance, *F_future participation_*(1,127) = 4.72, *p* < .05, *η_p_^2^* = .04, *F_difficulty level_*(1,127) = 60.96, *p* < .001, *η_p_^2^* = .32. Students who failed at the short-term memory task were significantly less eager to learn than students who succeeded. None of the other effects on the posttest eagerness to learn measures were significant.

## Discussion

In the present experiment, we examined how conditional regard impacts students’ self-esteem, affect and eagerness to learn following success or failure. Participants’ state self-esteem and positive affect decreased and negative affect increased after failure, whereas the reverse occurred after success. Also, failing students were less eager to learn than successful students. More importantly and in line with our hypotheses based on indirect evidence from previous research (e.g., Baldwin & Sinclair, 1996; Brummelman et al., 2014), we found that conditional regard magnifies success- and failure-induced changes in positive affect. When the regard of the experimenter was conditional, students who experienced success showed an increase in positive affect, whereas no significant change occurred when regard was not related to performance. Those who experienced failure showed greater decreases in positive affect when regard was conditional as compared to when it was not related to performance. Thus, conditional regard increases positive emotional experiences in the face of success, but it also dampens positive emotional experiences in the face of failure. As dampened positive affect has been identified as a risk factor for the development of depression (Raes, Smets, Nelis, & Schoofs, 2012), it may be hypothesized that, in this way, conditional regard may set the stage for emotional maladjustment and psychopathology. This is in line with and extends previous correlational research attesting to the negative correlates of conditional regard in achievement contexts (e.g., Assor & Tal, 2012; Roth et al., 2009).

Of note, these findings were specific to positive affect, and did not emerge for negative affect, state self-esteem, and eagerness to learn, thus yielding only partial support for our hypotheses. Possibly, state self-esteem and negative affect are more stable and, hence, more difficult to change based on a single statement signaling conditionality. Perhaps longer-term, repeated exposure to conditionality is needed for such effects to emerge. In the current study, stability coefficients were indeed higher for state self-esteem and negative affect as opposed to positive affect. Also, it may be possible that effects would have generalized to more stable outcomes, such as self-esteem, when the feedback would have been given by a significant other (e.g., the teacher) or when the feedback was given in a high-stake performance context (e.g., admission tests). Further research should thus investigate under which circumstances conditional feedback can augment performance effects on these outcomes too, by varying the significance of the person that gives feedback or by varying the performance context.

Even though results for positive affect did not extend to any of the other outcomes, the present study findings are important as they may inspire further research as well as provide a basis for more fine-grained hypotheses. Overall, our findings portray CR as a double-edged sword, showing that it intensifies increases in positive affect following success, but also decreases in positive affect following failure. Given that no one can be successful all the time (Assor et al., 2004), the net long-term effect of providing CR is likely to be negative.

Our study thus extends research into the consequences of CR, but there are also some limitations. First, sample size did not allow us to examine potential moderators and may also have limited power to detect small effects. Also, our sample mainly consisted of female psychology students. Future studies should use more diverse samples and establish generalizability. Second, we only looked at short-term effects on well-being and eagerness to learn. Within ethical boundaries, future studies may focus on the longer-term consequences. Third, in this experiment success and failure were manipulated between – and not within – subjects. Although the choice for a between-subjects design has a number of advantages, such as the prevention of possible carryover effects (Charness, Gneezy, & Kuhn, 2012), a within-subjects design may enable students to experience conditionality even more vividly by letting them both succeed and fail in subsequent tasks and varying regard accordingly. Finally, it may be interesting to replicate our results in an experimental design with CR coming from significant others to enhance external validity. Possibly, the effects of CR are stronger when expressed by significant others (friends, parents or teachers) with whom students have a personal bond, and such findings may also be translated more easily into tailored preventive interventions regarding CR.

Despite these limitations and going beyond previous survey research by using an experimental design, our study is the first to directly show that conditional regard intensifies changes in students’ emotional experience following success and failure.

## Author Information

This research was supported by a Postdoctoral Fellowship granted by the Research Foundation – Flanders (FWO) to the first author from 2014 to 2017 and by the KU Leuven Fund for Fair Open Access. The authors would like to thank Annabel Bogaerts and Evi Imler for their assistance in this research as part of their master’s thesis in psychology.
